# Modelling and Prediction of Cutting Temperature in the Machining of H13 Hard Steel of Transient Heat Conduction

**DOI:** 10.3390/ma14123176

**Published:** 2021-06-09

**Authors:** Jingjie Zhang, Xiangfei Meng, Jin Du, Guangchun Xiao, Zhaoqiang Chen, Mingdong Yi, Chonghai Xu

**Affiliations:** 1 School of Mechanical and Automotive Engineering, Qilu University of Technology (Shandong Academy of Sciences), Jinan 250353, China; 17862963084@163.com (X.M.); dj84105@126.com (J.D.); xgc@qlu.edu.cn (G.X.); czq@qlu.edu.cn (Z.C.); new-raul@163.com (M.Y.); xch@qlu.edu.cn (C.X.); 2 Key Laboratory of Equipments Manufacturing and Intelligent Measurement and Control, China National Light Industry, Qilu University of Technology (Shandong Academy of Sciences), Jinan 250353, China; 3 School of Materials Science and Engineering, Shandong University, Jinan 250000, China

**Keywords:** cutting temperature, coated tool, Laplace transform, non-Fourier heat conduction, transient heat conduction

## Abstract

Cutting heat conduction undergoes three stages that include intensity transient-state, transient-state, and steady-states. Especially during machining with coated cutting tools, in the conduction process, cutting heat needs to pass through a few micron thick coatings and then flow into the tool body. This heat conduction presents typical non-Fourier heat conduction characteristics. This paper focuses on the cutting temperature in transient heat conduction with a coated tool. A new analytical model to characterize the thermal shock based on the non-Fourier heat conduction was proposed. The distribution of cutting temperature in mono-layer coated tools during the machining was then illustrated. The cutting temperature distribution predicted by the Fourier heat conduction model was employed to compare with that by non-Fourier heat conduction in order to reveal the non-Fourier heat conduction effect in transient heat conduction. The results show that the transient heat conduction analytical model is more suitable for the intensity transient-state and transient-state in the process of cutting heat conduction.

## 1. Introduction

In recent years, more and more coated tools are employed in metal cutting applications, especially in the machining of difficult-to-machine materials. The application of a tool coating improves the wear resistance and hardness of the tool [1,2]. The thin film coated on the cutting tool surface can enhance the tool strength and decrease the friction between tools and workpieces, and it plays an important role in the process of heat conduction into the cutting tool body. The study of temperature distribution in the tool’s body is essential for the investigation of the thermal effect on tool life and workpiece quality. The heat generation and heat conduction of coated tools during the cutting process are very different compared with uncoated tools owing to the presence of the tool coating film. The low friction coefficient of the tool coating can reduce the cutting force and cutting temperature. The tool coating can also be used as a heat-resistant material for cutting tools to prevent excessive heat entering the tool matrix. Therefore, coated tools are widely used in machining [3], and the coating can obviously prolong a tool’s life.

Heat conduction during the cutting process can be divided into three states, including intensity transient-state, transient-state, and steady-state. Many researchers used Fourier heat conduction to study the cutting temperature in steady-state heat conduction. Sijie Yan et al. [4] presented a thermal model to describe the coated tool temperature variation in dry milling of nickel-based super alloys for a turbine blade and built a model by the steady-state heat conduction differential equations. In the proposed model, both the heat fluxes into the tool from the rake face and due to flank wear are calculated to estimate the tool temperature distribution at different tool states. The influence of flank wear is considered according to the rapid tool wear. Baïri et al. [5] studied the effect of coating materials on the thermal behavior of a body subjected to multiple moving heat sources. They calculated steady-state temperature using the finite complex Fourier transform and the finite cosine Fourier transform. Benabid et al. [6] presented a new procedure to evaluate the cutting temperature for milling operation. The predicted temperature from the presented analytical model is compared with the FEM both numerically determined and the experimentally determined temperatures at the same boundary condition. The compared results showed that the temperatures from analytical prediction, FEM simulation, and experimental measurement are in good agreement.

The evolution of the cutting temperature is well-described by the Fourier heat conduction in steady-state heat conduction. However, when heat conduction is fast and transient, some precautions are needed to carry out the numerical computation [7,8]. In the transient heat conduction of high heat flow, the time of thermal action is often less than the thermal relaxation time of the material, so heat conduction is heat propagating at a finite speed. When some points display thermal disturbance in the heat-conducting medium, the other points in heat-conducting medium will be affected simultaneously by the thermal disturbance. According to physical law, the speed of heat transmission cannot be faster than the speed of light. It is not acceptable that the assumption of thermal disturbance velocity is infinite. For the unsteady-state of the heat conduction process, such as high (or low) temperature conditions in heat conduction, a super high velocity of heat conduction, and under micro space or a very short time scale of heat conduction, the temperature changes rapidly. The assumption of Fourier heat conduction, which implies the heat conduction velocity is infinite, has not been suitable for the unsteady-state heat conduction. Mozafarifard et al. [9] aims to carry out the fast-transient process of heat flow in a porous medium, proposing the Caputo-type fractional Cattaneo sub diffusion model to study the anomalous diffusion process in the temperature response of the two-phase system. The classical Fourier model fails to interpret such effects because it is assumed that the thermal wave propagates with infinite speed, and the thermal interactions between phases occur simultaneously. The fractional time differentiation term can interpret the memory effects of the physical problem, providing the most accurate prediction of the transient process of heat flow in a solid–fluid system under the short thermal perturbation. Zehnder [10] observed the thermal wave phenomenon in the AISI4340 carbon steel through infrared temperature measurement. The experimental results showed that the temperature increases were high over ambient and the region of intense heating would not be able to apply the traditional Fourier heat conduction. The non-Fourier heat conduction in micro or nanopascal heat conduction for metal materials modified by the classical Fourier heat conduction should be taken into consideration in the cutting intensity transient-state.

In the 1960s, Cattaneo [11] and Vernotte [12] proposed the non-Fourier heat conduction independently. Its mathematical expression is hyperbolic partial differential equations. For the abnormal state Fourier heat conduction effect, the characteristics of non-Fourier heat conduction effect were given, i.e., a very short time of a thermal effect, thermal heating material small or medium scale, or completely deviating from the classical Fourier heat conduction effect. As the hyperbolic heat conduction equation has been proposed, many researchers tried to solve it in a variety of different physical conditions, developing a variety of mathematical methods to accurately predict different physical geometry and boundary conditions for the temperature field [13,14]. Schwarzwlde et al. [15] have studied non-classical heat transport in phase-change processes as described by the Maxwell–Cattaneo (MC) and Guyer–Krumhansl (GK) equations via the solidification kinetics of a one-dimensional liquid bath.

Numerous approaches have been proposed to model the metal cutting temperature of coated tools, with various degrees of success. Along with complicated experimental approaches, several analytical and numerical techniques have been proposed to model the temperature distribution. Ferreira et al. [16] used numerical analysis of the influence of coatings on a cutting tool. The coating fulfilled its role of protecting the substrate of the cutting tool with respect to heat. Zhang et al. [17] used an analytical model to study the effect of coating materials in transient heat conduction and found that the coating has a thermal barrier property in heat conduction during machining. This paper describes a new approach for predicting cutting temperature distribution in transient heat conduction of monolayer coated tools based on non-Fourier heat conduction. The characteristics for temperature distribution in unsteady-state and steady-state are compared and discussed to illustrate the existence of the non-Fourier heat conduction effect in the transient-state. The temperatures have a fluctuating change process, which is observed by temperature distribution of a TiN-coated tool during transient heat conduction.

## 2. Modeling of Heat Conduction in Coated Tools

**Figure 1:**
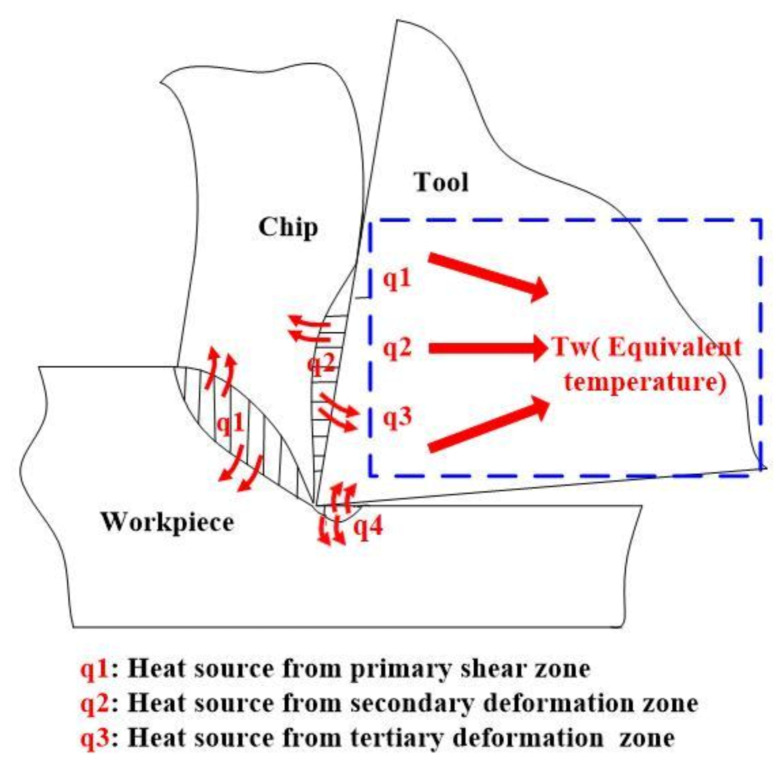
Heat source and equivalent heating temperature of the cutting tool.

In the cutting process, heat generation occurs at the shear plane by primary deformation, tool–chip interface, and tool–workpiece interface by friction, as shown in Figure 1 [18]. The generated heat is concentrated on the region around the cutting edge. However, the value of heat flux on the rake face is difficult to obtain through the measurement or calculation method owing to the very small space of the three heat sources and very short cutting time. For simplicity, it is assumed that there is no temperature gradient in the rake face, and there is no heat source and no heat generation in the tool body. The generated heat can be taken as one equivalent constant temperature acted on the rake face of coated tool in one-dimensional heat conduction, as shown in Figure 1.

**Figure 2:**
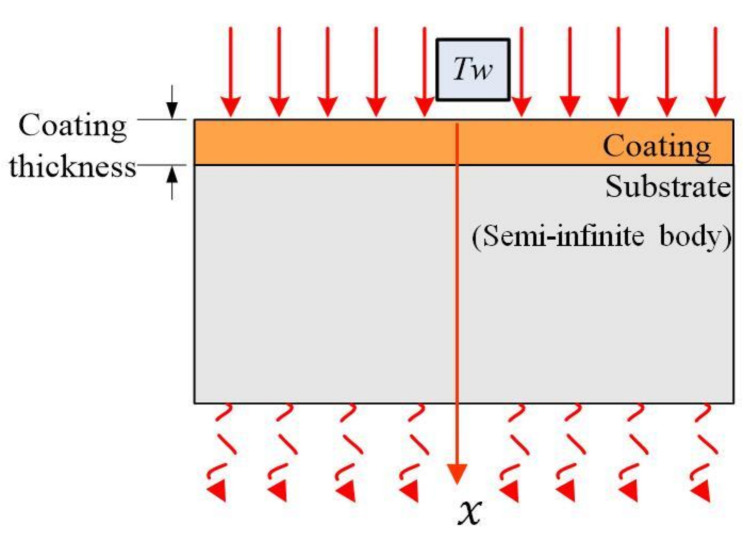
Schematic of one-dimensional heat conduction model of coated tools.

The coating thickness of the mono-layer coated tools is generally very thin, between 1 and 8 μm [19] When the tool cuts into the work piece, the tool coating is subjected to strong instantaneous thermal shock. With the increase of cutting time, the transient degree gradually weakens, and finally transits to steady-state heat conduction. The coated tool substrate is sufficiently large compared with mono-layer coating and can then be considered to be a semi-infinite body. Figure 2 shows a schematic of heat conduction model for a one-dimensional coated tool. For the heat conduction calculation, the following assumptions were used:

(1) Machining is performed at ambient temperature assuming that the initial temperature of both the workpiece and the tool is equal to the room temperature (*T*_0_ = 20 °C).
(2) The heat flux into the coated tools will pass through the thin coating layer and the substrate of the tools and will not be lost through other ways. There is not any heat conduction with the environment.

For the modeling of proposed unsteady-state heat conduction, the following assumptions are made to derive the mathematical modeling of the proposed unsteady-state heat conduction process.

(1) Thermal properties such as conductivity and diffusivity are independent of temperature, and they are uniform for a coating layer.
(2) The substrate body of the cutting tool is a semi-infinite body along the coating thickness direction as shown in Figure 2.

Based on the established one-dimensional heat conduction model of the coated tools, the unsteady-state heat conduction model of the mono-layer coated tool is established by the non-Fourier law of heat conduction. *X* direction is the cutting heat conduction direction. *T(x*, *t)* is the transient temperature at point *(x*, *t)*, and it is a continuous function of space coordinates *x* and time coordinates *t*. It is assumed that environment temperature is *T*_0_ in the cutting process. When the cutting time is *t* = 0^+^, the rake face of coated tool is shocked by an equivalent constant temperature *T_w_* (*T_w_* > *T*_0_).*T_w_* is assumed to always act on the top surface of the coating and will remain constant during the heat conduction process. *τ* is the relaxation time, *a* is the thermal diffusivity of the coating material, *q* is the heat flux, *d* is the coating thickness, and *L* is the coated tool thickness. According to the above, it is assumed that the heat is only conducted along the direction of coating thickness. The mathematical model to describe the temperature field of *T(x*, *t)* in the coating using non-Fourier heat conduction is given as follows.

(1) $$a\frac{\partial^{2}T}{\partial x^{2}}=\tau_{0}\frac{\partial^{2}T}{\partial t^{2}}+\frac{\partial T}{\partial t}\;\;\;0<x<d,\;\;t\;\geq \;0$$

Initial conditions:

(2) $$T\left(x,t\right){|}_{t=0}=T_{0}\;\;\;\;0\;\leq \;x\;\leq \;d\;\frac{\partial T\left(x,t\right)}{\partial t}{|}_{t=0}\;0\;\leq \;x\;\leq \;d$$

Boundary conditions:

(3) $$T\left(x,t\right){|}_{x=0}=T_{w}t\geq 0\;\frac{\partial T\left(x,t\right)}{\partial t}{|}_{x=d}t\geq 0$$

The illustration of the initial conditions is the temperature of the cutting tool remains at *T*_0_ before heat load effects at the boundary, and it is in a natural state. The boundary condition is that the coated tool is an adiabatic boundary during heat conduction.

To introduce the excess temperature $T^{*}$,

(4) $$T^{*}\left(x,t\right)=T\left(x,t\right)-T_{0}$$

Then, the heat conduction control equation, initial conditions, and boundary conditions become the following:

Heat conduction control equations:

(5) $$a\frac{\partial^{2}T^{*}}{\partial x^{2}}=\tau_{0}\frac{\partial^{2}T^{*}}{\partial t^{2}}+\frac{\partial T^{*}}{\partial t}\;\;\;0<x<d,\;\;t\;\geq \;0$$

Initial conditions:

(6) $$T^{*}\left(x,t\right){|}_{t=0}=0\;\;\;0\;\leq \;x\;\leq \;d\;\frac{\partial T^{*}\left(x,t\right)}{\partial t}{|}_{t=0}\;\;\;0\;\leq \;x\;\leq \;d$$

Boundary conditions:

(7) $$T^{*}\left(x,t\right){|}_{x=0}=T_{w}\;\;\;\;t>0\;\frac{\partial T^{*}\left(x,t\right)}{\partial t}{|}_{x=d}=0\;\;\;t>0$$

Equation (5) is conducted to a Laplace transform:

(8) $${\bar{T}}^{*}\left(x,s\right)=L\left[T^{*}\left(x,s\right)\right]=\int_{0}^{\infty }T^{*}\left(x,t\right)e^{-st}dt$$

(9) $$a\frac{\partial^{2}{\bar{T}}^{*}}{\partial x^{2}}\left(x,s\right)=s{\bar{T}}^{*}\left(x,s\right)-T^{*}\left(x,0\right)+\tau \left[s^{2}{\bar{T}}^{*}\left(x,s\right)-sT^{*}\left(x,0\right)-\frac{\partial }{\partial t}T^{*}\left(x,0\right)\right]$$

The initial conditions (6) into Equation (9) are as follows:

(10) $$a\frac{\partial^{2}{\bar{T}}^{*}}{\partial x^{2}}\left(x,s\right)=\left(s+\tau s^{2}\right){\bar{T}}^{*}\left(x,s\right)$$

The solution of Equation (10) is as follows:

(11) $${\bar{T}}^{*}\left(x,s\right)=A\left(s\right)e^{-\sqrt{\frac{s+\tau s^{2}}{a}}x}+B\left(s\right)e^{\sqrt{\frac{s+\tau s^{2}}{a}}x}$$

The boundary conditions (7) are obtained by Laplace transform:

(12) $${\bar{T}}^{*}\left(x,s\right){|}_{x=0}=\frac{T_{w}-T_{0}}{s}\;\frac{\partial {\bar{T}}^{*}\left(x,t\right)}{\partial t}{|}_{x=d}=0$$

Substituting Equation (12) into Equation (11) gives the following:

(13) $$A\left(s\right)+B\left(s\right)=\frac{T_{w}-T_{0}}{s}$$

(14) $$A\left(s\right)e^{-\sqrt{\frac{s+\tau s^{2}}{a}}x}+B\left(s\right)e^{\sqrt{\frac{s+\tau s^{2}}{a}}x}=0$$

From Equations (13) and (14),

(15) $$A\left(s\right)=\frac{T_{w}-T_{0}}{s}\times \frac{1}{1-e^{-2\sqrt{\frac{s+\tau s^{2}}{a}}d}}$$

(16) $$B\left(s\right)=\frac{T_{w}-T_{0}}{s}\times \frac{1}{1+e^{2\sqrt{\frac{s+\tau s^{2}}{a}}d}}$$

Substituting Equations (15) and (16) into Equation (11) gives the following:

(17) $${\bar{T}}^{*}\left(x,s\right)=\frac{T_{w\;}-T_{0}}{s}\times \frac{1}{1-e^{-2\sqrt{\frac{s+\tau s^{2}}{a}}d}}e^{-\sqrt{\frac{s+\tau s^{2}}{a}}x}+\frac{T_{w}-T_{0}}{s}\times \frac{1}{1-e^{2\sqrt{\frac{s+\tau s^{2}}{a}}d}}e^{\sqrt{\frac{s+\tau s^{2}}{a}}x}$$

Laplace transform is performed on Equation (17):

(18) $$T^{*}\left(t\right)=\frac{ln2}{t}\overset{N}{\underset{i=1}{\sum }}C_{i}T^{*}\left(\frac{ln2}{t}i\right)$$

(19) $$C_{i}={\left(-1\right)}^{i+N}\times \overset{\min\left(i,\frac{N}{2}\right)}{\underset{k=int\left[\left(i+1\right)/2\right]}{\sum }}\frac{k^{\frac{N}{2}}\left(2k\right)!}{\left(\frac{N}{2}-k\right)!k!\left(k-1\right)!\left(i-k\right)!\left(2k-i\right)!}$$

Because $T^{*}=T-T_{0}$, so $\;T=T^{*}+T_{0}$.

Finally, the following is concluded:

(20) $$T\left(t\right)=\frac{ln2}{t}\overset{N}{\underset{i=1}{\sum }}C_{i}T^{*}\left(\frac{ln2}{t}i\right)+T_{0}$$

Equation (20) is a transient heat conduction model of coating tool cutting heat derived based on non-Fourier heat conduction.

## 3. Experimental Setup

**Figure 3:**
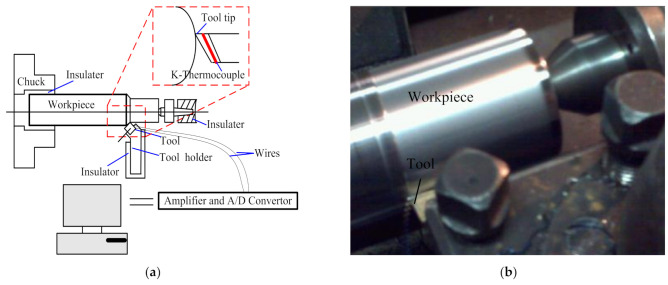
Tool temperature measurement set-up (**a**) schematic and (**b**) actual photograph.

In order to verify the analysis model, thermocouples are used to measure the cutting temperature of coated tools in the cutting process. The work piece material used in the turning experiment is H13 hardened steel. A general overview of the experimental setup is shown in Figure 3a. Photos of the experimental set–up are shown in Figure 3b. The workpiece employed in this experiment was a cylindrical bar with 300 mm length and 70 mm external diameter. The cutting speeds were *v_c_* = 35.9 m/min, 56.1 m/min, 89.2 m/min, 110.7 m/min, 197.8 m/min, and 244.4 m/min, respectively. The feed rate was *f* = 0.2 mm/rev, while the depth of cut was *a_p_* = 0.2 mm. The machining operation was then performed with a constant chip section and a constant cutting speed.

**Figure 4:**
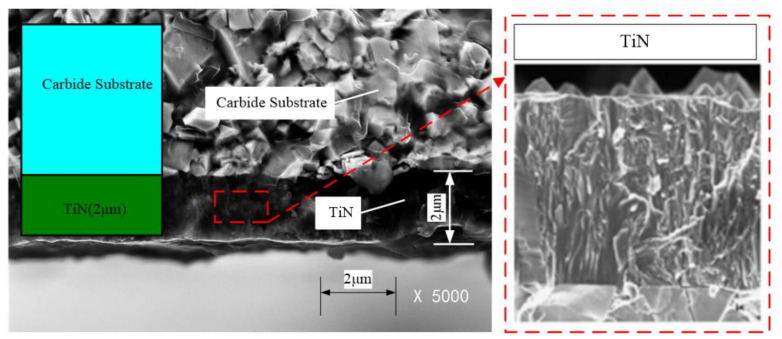
Structure of carbide substrate and components of KT315 coated cutting tool. Magnification: 5000×.

The tool is a mono layer coated tool, namely, KC735M cutting tool (Kennametal Inc., Latrobe, PA, USA) with TiN coating layer of carbide substrate. The thickness of the coating layer is 2 μm, given by the manufacturer—KENNAMETAL. A standard Scanning electron microscope (SEM, Carl Zeiss AG, Haute-Cohen, Germany) at a fracture cross-section of the coated sample was given to the thickness measurements shown in Figure 4.

Experimental work was carried out to measure the cutting tool temperature by developing wireless temperature measurement. The thermocouple sensor is embedded in the turning tool. The tool drilled two mounting holes to install the thermocouples sensor. The mounting hole is 2 mm distant from the tool tip. The depth of mounting hole is 1 mm, and the diameter is 1 mm. High temperature structure adhesive HT–CPS covered the top of the sensor to prevent damage by the cutting chips. Electric voltage converted by temperature difference is amplified. The electric signals can be translated from the sensor to computer through the amplifier and A/D convertor.

## 4. Results and Discussion

According to the transient heat conduction model, the computing process is programmed in MATLAB (Version 2017b, MathWorks.Inc, Natick, MA, USA). The equivalent temperature of rake face is assumed to be *Tw* = 300 °C. The thickness of the coating layer is selected as *d* = 2 μm. The physical properties of TiN coatings and H13 steel are shown in Table 1.

### 4.1. Experimental Verification

**Figure 5:**
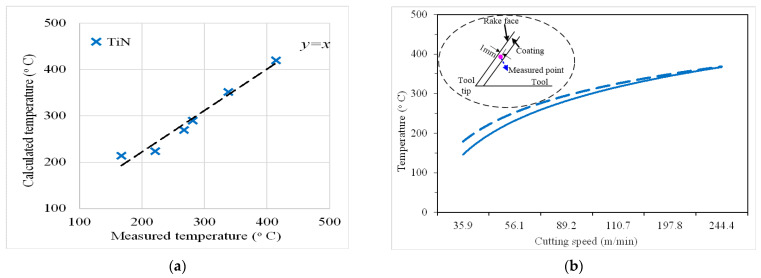
The temperature distribution of TiN-coated tools: (**a**) relationship between measured and calculated values and (**b**) measured and calculated values versus cutting speed.

Figure 5 illustrates the cutting temperature distribution obtained from the analytical model and the thermocouple measurement, respectively. The calculated temperature is the temperature at the thermocouple measurement point, which is 1 mm distance from rake face. From Figure 5, the measured and calculated temperatures present a linear relationship; the function is *y = x*. The values’ trends obtained through calculation and measurement show good overall consistency. According to the transient analysis model, the calculated temperature of thermocouple measurement point showed the similar trend of measured temperature in the same measured point. The calculated and measured temperature profiles are plotted at different cutting speeds, as shown in Figure 5b.

In the modeling process, the analytical model is simplified and contained hypothesis conditions. When the convective heat conduction coefficient of the boundary is very small or the heat conduction coefficient is very small, the boundary is simply defined as an adiabatic boundary or a thermostatic boundary. The heat dissipation of the coated tool and workpiece material during the actual cutting process is ignored. The prediction error is less than 12%, which is an acceptable level for the industry applications and proves the effectiveness of the analytical model.

### 4.2. Effects of Cutting Time on Temperature Distribution

**Figure 6:**
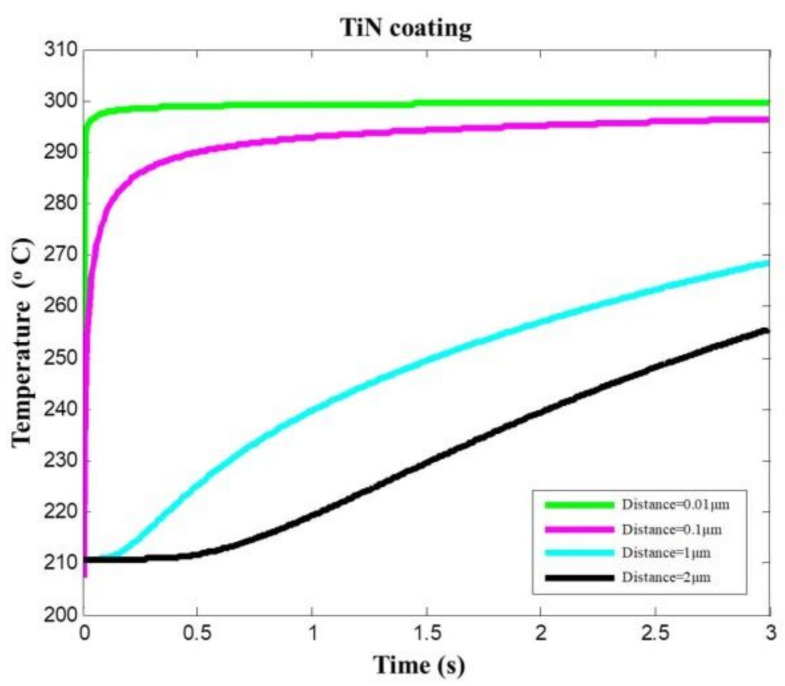
Temperature prediction at different locations within the TiN-coated tool coating.

**Figure 7:**
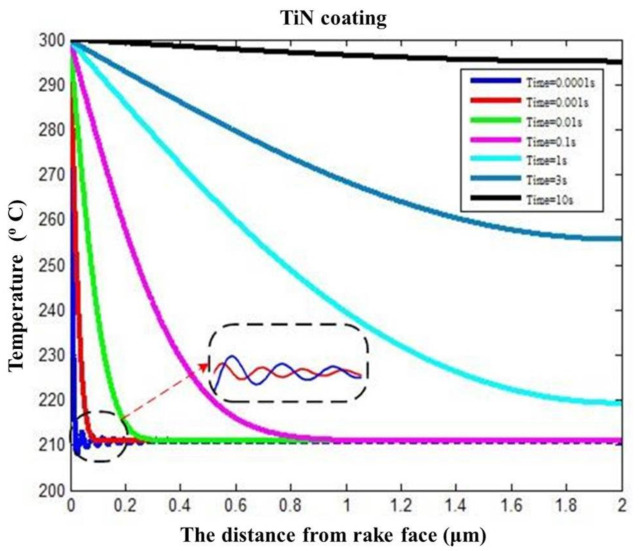
Temperature prediction in TiN coating changing over distance from coating surface.

According to the transient heat conduction model, the cutting temperatures of TiN-coated tools are calculated, as shown in Figure 6 and Figure 7. In Figure 6, in 0.5 s cutting time, the temperature of coating surface increases from the initial temperature 20 °C to the assumed equivalent temperature of 300 °C (0.01 μm from the coating surface); the temperature of the coating–substrate interface increases from 20 °C to close to 210 °C (2 μm from the coating surface). Different positions inside the coating have different temperature values at the same time. When the cutting time of 2 s is achieved, the coating surface temperature (0.01 μm from the coating surface) is higher than the coating body temperature (0.1 μm from the coating surface). At the same cutting time, the farther the distance from the coating surface, the lower the temperature. This indicates that the cutting heat conduction is a finite velocity heat conduction.

The influence of cutting time (0.0001, 0.001, 0.01, 0.1, 1, 3, and 10 s) on temperature distribution was investigated. The cutting temperature variation at different cutting times shows a decreasing trend from 300 °C (i.e., the boundary temperature) as the distance from the coating surface increases. It is observed from Figure 7 that the difference values between the maximum temperature (i.e., the coating surface temperature) and the minimum temperature (i.e., the distance from the coating surface is 2 μm and the coating–substrate interface temperature) decrease with the cutting time. The heat conduction states can be recognized from the variations of cutting temperature over cutting time. The intensity transient heat conduction occurs when the cutting time in 1 s. When the cutting time is between 1 s and 10 s, there is transient heat conduction, and the thermal disturbance is not obvious. When the cutting time is >10 s, it is a gradual heat conduction process from transient-state to steady-state, and the temperature change is relatively small.

The temperature shows a rapid reduction and delays the change process in the heat conduction process within a very cutting short time, namely, in the unsteady-state heat conduction phase (i.e., blue and red curves in Figure 7). The temperature fields indicated by the blue and red curves were rapidly reduced to a certain temperature (210 °C) and presented a fluctuating change trend (the partial view as shown in Figure 7). The temperature fields sometimes are higher than the certain temperature, and sometimes are less than the certain temperature. The temperature calculation with transient heat conduction analytical model shows that the temperature changes are a delay rapid growth process. The fluctuation of the red curve is a not obvious owing to the decreased transient degree. The fluctuations of the subsequent curves disappear gradually, as the heat conduction states changes from the intensity transient-state to transient-state and then to steady-state as the cutting time increases.

### 4.3. Temperature Distribution with Fourier and Non-Fourier Heat Conduction

**Figure 8:**
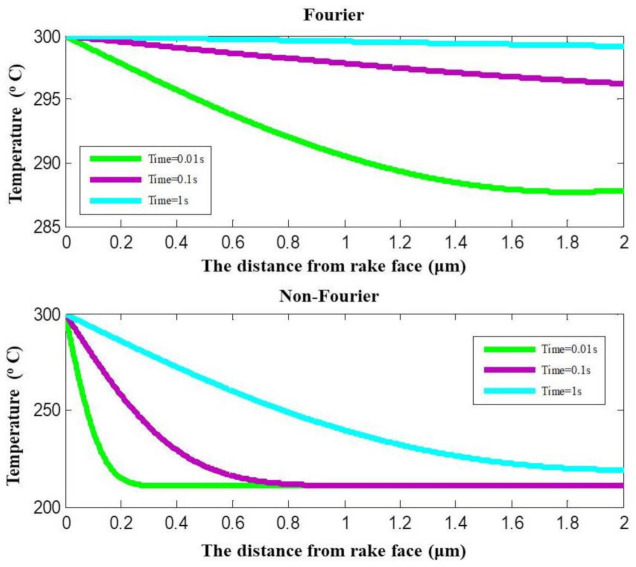
Temperature prediction with Fourier and non-Fourier heat conduction.

It can be observed from Figure 8 that the temperatures predicted by Fourier heat conduction and non-Fourier heat conduction at the same cutting time and the identical location are in great difference. The temperature calculated with Fourier heat conduction does not present a thermal disturbance at intensity transient heat conduction. It shows a reduction process starting from the boundary temperature of 300 °C. The temperature calculated with non-Fourier heat conduction has a rapid reduction in unsteady-state heat conduction. Then, the temperature goes through a fluctuation change process. With the reduction of the transient degree, the fluctuation changes disappear gradually and present a temperature reduction.

**Figure 9:**
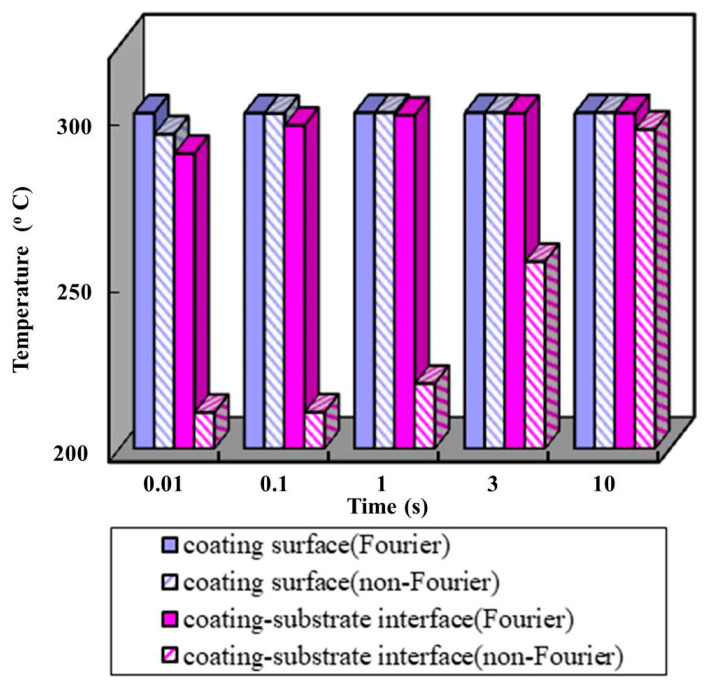
Temperatures prediction at coating surface and coating–substrate interface.

The temperatures at coating surface and coating–substrate interface calculated with non-Fourier heat conduction and Fourier heat conduction are shown in Figure 9, respectively. The predicted temperature at the coating–substrate interface is very different between the two models. When the cutting time is 0.01 s, the calculated temperature difference between the two models is 77.1 °C. When the cutting time is 10 s, the calculated temperature difference between with the two models is 4.9 °C. When the cutting time is long enough and heat conduction is at the steady-state, the temperature tends to stabilize. The cutting heat conducts through the thin film coating on the tool surface to the inside of the tool can be described a transient heat conduction with a finite velocity.

## 5. Conclusions

In this paper, the cutting temperature of mono-layer coated tool in the unsteady-state and steady-state was studied. Based on the analytical models, the cutting temperatures of TiN-coated tools under different heat conduction states were calculated. The main conclusions are as follows:

1. The effects of cutting time on TiN-coated tools’ temperature distribution were investigated. When cutting time was short enough, the temperatures have a fluctuating change process, and the fluctuating change gradually disappears with the decreases with the weakening of the transient degree. With the increase of cutting time, the heat conduction changed from transient-state to steady-state, the cutting temperature increased, and stabilized gradually. When the cutting time is constant, the farthest away from the coating surface, the lower the temperature in the tool body.
2. It is found that the non-Fourier heat conduction effect exists in the cutting heat transient conduction of coated tool machining. When the heat conduction is transient heat conduction, the thermal disturbance and thermal delay caused by thermal shock can be accurately described by the non-Fourier heat conduction model.
3. The temperature predicted error with the transient heat conduction model is less than 12%. The transient heat conduction model was a suitable application to intensity transient-state and transient-state.

## Figures and Tables

**Table 1:** Physical properties of TiN coating and H13 [1,20].

Material	Thermal Conductivity λ (W/m·°C)	Thermal Diffusivity a (m^2^/s)	$\mathrm{Density}\;\rho \;\;(\mathrm{kg}/m^{3})$	Young’s Modulus *E* (Gpa)
TiN coating	23	5.07 × 10^−7^	4650	250
H13 steel	28.6	7.92 × 10^−6^	7800	211

## Data Availability

The datasets used or analyzed during the current study are available from the corresponding author on reasonable request.
